# Predictive Factors of Lapatinib and Capecitabine Activity in Patients with HER2-Positive, Trastuzumab-Resistant Metastatic Breast Cancer: Results from the Italian Retrospective Multicenter HERLAPAC Study

**DOI:** 10.1371/journal.pone.0156221

**Published:** 2016-05-25

**Authors:** Stefania Gori, Alessandro Inno, Valentina Rossi, Monica Turazza, Elena Fiorio, Alessandra Fabi, Giancarlo Bisagni, Jennifer Foglietta, Daniele Santini, Ida Pavese, Arianna Pellegrino, Alberto Zambelli, Patrizia Vici, Vita Leonardi, Sandro Barni, Silvana Saracchini, Giuseppe Bogina, Fabiana Marchetti, Simona Duranti, Gianluigi Lunardi, Filippo Montemurro

**Affiliations:** 1 Medical Oncology, Sacro Cuore Don Calabria Hospital, Negrar, Verona, Italy; 2 Medical Oncology, Ospedale Civile di Saluzzo, Saluzzo, Italy; 3 Medical Oncology, AO Universitaria Integrata Verona, Verona, Italy; 4 Medical Oncology A, INT Regina Elena, Roma, Italy; 5 Medical Oncology, IRCCS AO S.Maria Nuova, Reggio Emilia, Italy; 6 Medical Oncology, S.Maria della Misericordia Hospital, Perugia, Italy; 7 Medical Oncology, Università Campus Biomedico, Roma, Italy; 8 Medical Oncology, San Pietro Fatebenefratelli Hospital, Roma, Italy; 9 Medical Oncology, IRCCS Fondazione S. Maugeri, Pavia, Italy; 10 Medical Oncology B - INT Regina Elena, Roma, Italy; 11 Medical Oncology, ARNAS Civico, Palermo, Italy; 12 Medical Oncology, AO Treviglio – Bergamo, Bergamo, Italy; 13 Medical Oncology, S.Maria degli Angeli Hospital, Pordenone, Italy; 14 Pathology, Sacro Cuore Don Calabria Hospital, Negrar, Verona, Italy; 15 Investigative Clinical Oncology (INCO), Fondazione del Piemonte per L'Oncologia, Candiolo Cancer Institute (IRCCS), Candiolo, Italy; University of Torino, ITALY

## Abstract

**Background:**

There are no validated predictive markers for lapatinib and capecitabine in patients with trastuzumab-resistant HER2 positive metastatic breast cancer.

**Methods:**

Data of 148 consecutive patients treated with lapatinib and capecitabine from March 2007 to December 2013 were collected from 13 Italian institutions. Estimates of progression-free survival (PFS) and overall survival (OS) were obtained with the Kaplan-Meier method and compared with logrank test. The association of clinicopathological variables and the outcome was studied by binary logistic regression analysis and Cox proportional hazard analysis.

**Results:**

At a median follow-up of 41 months, median PFS and OS were 7 and 21 months, respectively. Patents with a PFS longer than 7 months had a significantly longer OS, compared with patients with a PFS equal to or shorter than 7 months (36 vs 15 months; p<0.001). Multivariate analysis revealed the benefit of lapatinib-based therapy in terms of PFS and OS was significantly associated with time-to-progression (TTP) on prior first-line trastuzumab-based therapy. In particular, each additional month on first-line trastuzumab based therapy was associated with a reduction in hazard of progression and death after the initiation of lapatinib-based therapy of 2% and 4%, respectively.

**Conclusions:**

A longer TTP to first line trastuzumab seems to predict a prolonged PFS and OS with subsequent lapatinib and capecitabine.

## Introduction

Overexpression of the human epidermal growth factor receptor 2 (HER2), occurring in approximatively 20–25% of metastatic breast carcinomas, is associated with poor prognosis, shorter progression-free survival (PFS) and overall survival (OS) [1,2]. Over the past decade, the outcome of patients with HER2 positive metastatic breast cancer has improved due to the development of effective HER2-targeted therapies, including the monoclonal antibodies trastuzumab [3] and pertuzumab [4,5], the antibody-drug conjugate trastuzumab emtansine [6] and the tyrosine kinase inhibitor lapatinib [7].

Lapatinib is a dual inhibitor of HER2 and epidermal growth factor receptor (EGFR). In a pivotal phase 3 trial, 399 patients with HER2-positive, locally advanced or metastatic breast cancer who had progressed after treatment regimens including an anthracycline, a taxane and trastuzumab, were randomized to receive lapatinib and capecitabine or capecitabine alone [7]. The addition of lapatinib to capecitabine translated into a significant improvement of median time to progression (TTP) from 4.4 to 8.4 months (hazard ratio 0.49, p<0.001), with a trend toward longer overall survival (hazard ratio 0.87, p = 0.210) [7,8]. Based on these results, in 2007 lapatinib in combination with capecitabine was approved by US Food and Drug Administration for the treatment of HER2 positive metastatic breast cancer patients previously exposed to trastuzumab, anthracyclines and taxanes.

Other two randomized phase 3 clinical trials showed the efficacy of lapatinib in combination respectively with trastuzumab or with letrozole for the treatment of metastatic breast cancer. In one trial enrolling 219 patients with hormone receptor positive, HER2 positive metastatic breast cancer, the addition of lapatinib to first line letrozole significantly extended median PFS from 3 to 8.2 months [9]. In the other study, 296 heavily pretreated women with HER2 positive metastatic breast cancer were randomized to receive lapatinib alone or lapatinib and trastuzumab. The final analysis of the study demonstrated a significant advantage for patients treated with the combination of the two HER2-targeted agents compared with those treated with lapatinib alone, in terms of median PFS (11.1 vs 8.1 weeks) and OS (14 vs 9 months), thus supporting a dual HER2 blockade in patients with pretreated HER2 positive metastatic breast cancer [10,11].

Lapatinib has been the only HER2-targeted agent available for trastuzumab-resistant patients, until the superiority of trastuzumab emtansine over lapatinib and capecitabine was demonstrated by the results of the EMILIA trial, published in 2012. The EMILIA trial was a phase 3 trial which randomized 991 patients with HER2 positive, metastatic breast cancer who had previously been treated with trastuzumab and a taxane, to receive trastuzumab emtansine or lapatinib and capecitabine [6]. Patients in the trastuzumab emtansine arm as compared with those in the lapatinib and capecitabine arm, achieved significantly longer median PFS (9.6 versus 6.4 months) and OS (30.9 versus 25.1 months at the second interim analysis) [6].

The combination of lapatinib and capecitabine, however, still remains a valid option for the treatment of HER2 positive metastatic breast cancer in the third line setting and beyond [12], and it seems to be particularly beneficial for some patients. In fact, about 20% of patients treated with lapatinib and capecitabine are progression-free at 12 months [6,7]. Although several putative biomarkers have been actively investigated [13,14], at the present time there are no validated predictive factors for selecting patients who may achieve a long disease control with lapatinib and capecitabine.

We designed this retrospective study to investigate predictors of outcome in patients with HER2 positive metastatic breast cancer receiving lapatinib and capecitabine.

## Methods

### Study design

Clinical and pathological data of consecutive patients with HER2 positive, metastatic breast cancer treated with lapatinib and capecitabine from March 2007 to December 2013 were retrospectively collected from 13 Italian institutions, through a specifically developed web-based case report form system. Patients were considered eligible if they had histologically confirmed metastatic breast cancer with HER2 positive status, assessed by immunohistochemistry (with 3+ indicating positive status), in situ hybridization, or both, and if they were previously treated with trastuzumab in the adjuvant and/or metastatic setting. Response to treatment was reviewed locally according to the response evaluation criteria in solid tumors (RECIST v. 1.0). Clinical end-points were PFS and OS. PFS was defined as the time from starting therapy with lapatinib and capecitabine to disease progression or death from any cause. OS was defined as the time from starting therapy with lapatinib and capecitabine to death from any cause.

### Statistical analysis

Clinical and pathological characteristics were reported using descriptive statistics. Median follow-up time and its interquartile range (IQR) were estimated according to the Kaplan-Meier inverse method. Estimates of PFS and OS were calculated according to the Kaplan-Meier product-limit method. Survival curves were compared with the logrank test. The association of clinical and pathological variables associated with the outcomes of interest was studied by binary logistic regression analysis and Cox proportional hazard analyses, where appropriate. The variables considered were: menopausal status at initial diagnosis of breast cancer, stage at initial diagnosis (IV vs I-III), tumor grade (G1-2 vs G3), proliferation index (Ki67, <20% *vs* ≥20%), hormone-receptor co-expression, receipt of neoadjuvant or adjuvant chemotherapy, receipt of adjuvant trastuzumab, receipt of adjuvant radiotherapy, disease-free interval between the first diagnosis of breast cancer and the first diagnosis of metastasis, pattern of metastatic disease (visceral vs non visceral), duration of first-line trastuzumab-based therapy, number of prior treatments before lapatinib (dichotomized around the median value) and best response to first trastuzumab-based therapy (response + disease stabilization vs progressive disease). In each model we initially included all the variables considered, regardless of their univariate significance, and then selected the final model by backward stepwise elimination. Results are reported as odds ratios (OR) and hazards ratios (HR) with 95% confidence intervals (95% CI). Statistical analysis was performed using SPSS version 17.0 (SPSS Inc., Chicago, IL). Because of the exploratory nature of this study, we set statistical significance at p≤0.10.

### Ethics statement

The study protocol was reviewed and approved by the following Ethic Committees: Comitato Etico per la Sperimentazione Clinica delle province di Verona e Rovigo, Comitato Etico Centrale Sezione IRCCS—IFO—Fondazione G.B. Bietti, Comitato Etico provinciale di Reggio Emilia, Comitato Etico delle Aziende Sanitarie della Regione Umbria, Comitato Etico dell’Università Campus Bio-medico di Roma, Comitato Etico Lazio 1, Comitato Etico Centrale della Fondazione Salvatore Maugeri, Comitato Etico Palermo 2, Comitato Etico dell’Azienda Ospedaliera di Treviglio, Comitato Etico A.O.U. S. Luigi Gonzaga di Orbassano. Given the retrospective nature of the study, no specific written informed consent was required. Patients records were anonymized and de-identified prior to analysis.

## Results

From March 2007 to December 2013, 148 patients with HER2-positive metastatic breast cancer across 13 Italian institutions were enrolled in the study. Demographics and main clinical and pathological characteristics of the patients are reported in Table 1. Nationality was: Italian for 142 patients, Romanian for 2 patients, Albanian, Dominican, Greek and British for 1 patient. Median age at the diagnosis of metastatic disease was 52 years. *De novo* metastatic patients were 33 (22%). Seventy-two (49%) patients were post-menopausal. Ninety-four (64%) patients had a G3 tumor, proliferation index was elevated (i.e. Ki67 ≥ 20%) in 99 (67%) patients, estrogen and progesterone receptors were ≥ 10% positive in 92 (62%) and 66 (45%) patients, respectively. Adjuvant and/or neoadjuvant therapy was administered to 108 (73%) patients; 53 patients (36%) received adjuvant/neoadjuvant trastuzumab

**Table 1:** Characteristics of patients.

Characteristics	n = 148
**Nationality**	
Italian	142
Albanian	1
Dominican	1
Greek	1
Romanian	2
British	1
**Age at diagnosis**	
Median Age at first diagnosis of EBC, years (range)	49 (22–82)
Median Age at first diagnosis of MBC, years (range)	52 (22–84)
**Stage at diagnosis**	
I	7% (10)
II	38% (56)
III	32% (48)
IV	22% (33)
Unknown	1% (1)
**Hormone receptor status**	
ER ≥10%	62% (92)
PgR ≥10%	45% (66)
**Grading**	
1	1% (2)
2	22% (33)
3	64% (94)
Unknown	13% (19)
**Ki67 status**	
≥ 20%	67% (99)
< 20%	18% (27)
Unknown	15% (22)
**Menopausal status**	
Post	49% (72)
Pre	51% (76)
**Previous treatments**	
Prior adjuvant/neoadjuvant chemotherapy (n)	73% (108)
Prior exposure to anthracyclines (n)	66% (97)
Prior exposure to taxanes (n)	47% (70)
Prior exposure to adjuvant/neoadjuvant trastuzumab	36% (53)
Prior exposure to adjuvant endocrine therapy	43% (64)
Adjuvant radiotherapy (n)	52% (77)
Median number of therapy lines administered for MBC (range)	4 (1–10)
**Number of metastatic sites at first diagnosis of MBC**	
1	36% (53)
2	34% (50)
≥3	29% (43)
Unknown	1% (2)
**Sites of metastatic disease at first recurrence**	
Liver	37% (55)
Lung	28% (41)
Bone	47% (70)
Soft-tissue/nodes	54% (81)
Effusions	11% (16)
Central nervous system	18% (27)

Abbreviations: EBC: early breast cancer; MBC: metastatic breast cancer; ER: estrogen receptor; PgR: progesterone receptor.

Before receiving lapatinib, patients had received a median of 4 previous lines for the treatment of metastatic disease (range 1–10).

At the time of this analysis, 88 patients had died from cancer progression, 1 had died for other causes, 48 where alive and on anticancer treatments and 11 were lost to follow-up. Median follow-up for alive patients was 60 months (IQR 15–105 months) and 36 months (IQR 16–56 months) from the first diagnosis of metastatic disease and from the first dose of lapatinib, respectively. Median PFS and OS from the first dose of lapatinib were 7 months (95% C.I. 6–8 months) and 26 (95% C.I. 21–31 months) months, respectively.

**Fig 1:**
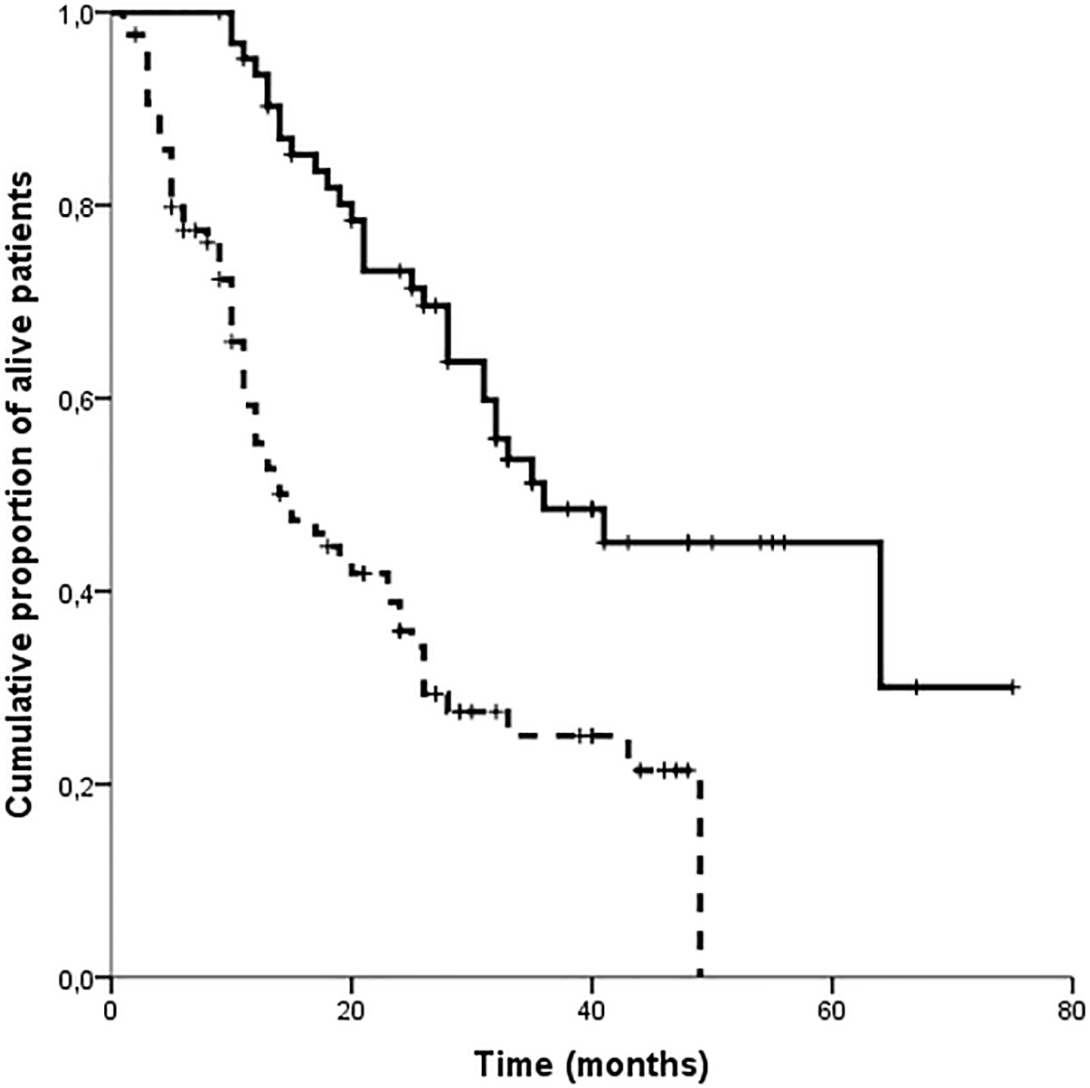
Overall survival. Kaplan Meier estimates of overall survival evaluated from the date of lapatinib-based therapy initiation in 132 patients according to PFS >7 months (solid line) or PFS ≤7 months (dashed line). Median overall survival is 36 months (95% C.I. 9–21 months) and 15 months (19–53 months), respectively (p<0.001).

To investigate the impact on OS of a prolonged disease control with lapatinib and capecitabine, we divided patients in 2 groups according to their PFS time, using the median PFS as the cut-off value. Patients with a PFS > 7 months were considered to have prolonged disease control with lapatinib and capecitabine, whereas patients with a PFS ≤ 7 months were considered to have a short disease control. Sixteen patients were not included in any of the two groups and therefore were excluded from the analysis. One patient was excluded because at the time of data cut-off was progression-free and still on treatment, but with a follow-up time shorter than 7 months. The other fifteen patients were excluded because they discontinued the treatment earlier than 7 months for reasons other than disease progression. Among the 136 patients included in the analysis, 63 patients achieved prolonged disease control (PFS > 7 months) and 69 patients had a short disease control (PFS ≤ 7 months). Median OS from the initiation of lapatinib-based therapy was significantly longer in patients with prolonged disease control (36 vs 15 months; p<0.001) (Fig 1).

We tried to investigate predictors of prolonged disease control with lapatinib-based therapy by logistic regression analysis but only best response to the initial trastuzumab-based therapy for metastatic disease was associated with this outcome. In particular, among the 128 patients with measurable disease, the 18 patients failing to respond to trastuzumab-based therapy had an about 64% reduction in the likelihood of a prolonged disease control with lapatinib-based therapy, compared with the 110 patients achieving objective response or disease stabilization with trastuzumab (Odds ratio 0.36, 95% C.I. 0.11–1.17, p = 0.09). In fact, 9 of these 18 patients (50%) experienced progressive disease (PD) at the first evaluation during lapatinib-based therapy, compared with 21 (19%) of those who had achieved response or disease stabilization to the first trastuzumab-based treatment (p = 0.01).

With the aim of further expanding the predictors of outcome, we concentrated on Cox Proportional Hazard models to study PFS and OS after the initiation of lapatinib-based therapy.

Multivariate analysis (Table 2) revealed that PFS was favorably influenced by having received less prior treatments for metastatic disease and by the duration of first-line trastuzumab-based therapy. In particular, TTP during first-line trastuzumab-based therapy was significantly associated with a 2% reduction in the hazard of progression for each additional month of therapy. OS after the initiation of lapatinib-based therapy was shorter in patients with visceral metastases compared with those without visceral involvement, and longer in Ki67 high (≥20%) or unknown tumors, compared with those with low Ki67 (<20%). Interestingly, and similarly to PFS, OS was better in patients with longer TTP to first-line trastuzumab-based therapy, with a 4% reduction in the hazard of death for any additional month of therapy.

**Table 2:** Multivariate analysis for Progression-free survival (PFS) and Overall Survival (OS) from the start of lapatinib-based therapy.

	PFS			OS		
Variable	HR	95% C.I.	P	HR	95% C.I.	P
Stage at initial diagnosis						
Pattern of disease						
Non-visceral	-	-	-	1	-	-
Visceral	-	-	-	1.84	1.10–3.07	0.02
Ki67						<0.01
<20%	-	-	-	1		
≥20%	-	-	-	0.34	0.19–0.60	<0.01
Unknown	-	-	-	0.47	0.23–0.94	0.03
Number of treatments before lapatinib						
≥4	1			-	-	-
<4	0.66	0.46–0.96	0.04	-	-	-
TTP to 1^st^ line trastuzumab (continuous)	0.98	0.97–1.00	0.01	0.96	0.95–0.98	<0.01

Abbreviations: HR, hazard ratio; PFS, progression-free survival; OS, overall survival; C.I., confidence interval; TTP, time to trastuzumab progression from the date of the first trastuzumab-based treatment for metastatic disease to the documented date of progression during or after treatment.

## Discussion

Trastuzumab and chemotherapy has been for years the mainstay of treatment of HER2 positive metastatic breast cancer. In the last few years, the landscape of therapy for HER2 positive metastatic breast cancer has rapidly evolved with the development of several effective HER2-targeted agents, with further improvement in OS with the use of new drugs. The addition of pertuzumab to first line trastuzumab and docetaxel impressively extended OS from 40.8 to 56.5 months [5]. Trastuzumab-emtansine as second line therapy in metastatic setting obtained a statistically significant longer PFS and OS compared with lapatinib and capecitabine, with an absolute benefit of 5.8 months in median OS [6]. The combination of lapatinib plus capecitabine still represents an option for HER2 positive breast cancer patients, especially in the third line and beyond [12]. In such evolving scenario, the research for predictive factors of response to HER2-targeted therapies is useful, in order to tailor the best therapeutic strategy to the individual patient.

In the present retrospective study, we evaluated 148 patients with HER2 positive metastatic breast cancer treated with lapatinib and capecitabine after the failure of at least one previous line of trastuzumab-containing therapy. In this ‘real life’ setting, we observed a median PFS of 7 months and a median OS of 21 months, which are comparable to those reported by clinical trials with lapatinib and capecitabine [7]. These results are particularly relevant, considering that in our study patients were heavily pretreated, with a median of 4 previous lines of treatment. This finding adds further data to the evidence of the activity of lapatinib and capecitabine in previously treated HER2 positive metastatic breast cancer patients.

We observed that patients who achieved a PFS longer than 7 months with lapatinib and capecitabine also derived a significant OS benefit when compared with patients who experienced disease progression within 7 months or earlier (36 vs 15 months; p>0.001). Even with the obvious limits of a retrospective analysis, these data suggest that the treatment with lapatinib and capecitabine may have a meaningful impact on survival for responding patients, and the investigation of markers predictive of prolonged disease control could help to identify patients who may derive a survival benefit. In the absence of validated predictive biomarkers, we investigated clinicopathological characteristics as predictive factors of prolonged disease control with lapatinib and capecitabine.

In our study, patients with visceral metastatic involvement before starting first line trastuzumab had higher risk of death on lapatinib and capecitabine. The detection of visceral metastases at the time of first line treatment is probably associated with a more aggressive biological behavior of the disease, thus representing a general prognostic factor rather than a predictive factor for lapatinib and capecitabine. Several retrospective analyses reported worse prognosis for patients with visceral metastases at the first relapse compared with patients with skeletal metastases only [15–17]. Not unexpectedly, we also found that receiving lapatinib-based therapy as earlier line of treatment after failure to trastuzumab was associated with better PFS.

The most interesting finding of our study, however, was that the performance of first line trastuzumab-based therapy was a predictor of clinical outcomes with lapatinib and capecitabine. Women failing to achieve at least SD to first-line trastuzumab had reduced PFS with lapatinib and capecitabine (<7 months) and, subsequently, shortened OS. In our study, the PD rate to first line trastuzumab-based therapy was 14% (18 out of 128 patients who were evaluable for response, 95% C.I. 8–20%). The rate of PD in clinical trials of chemotherapy (docetaxel or vinorelbine) with trastuzumab ranges between 5% to 8% [4,18]. Due to the retrospective nature of our study and lack of centralized revision of HER2 status, we could not confirm that all these non-responders had HER2-positive tumors. Despite this limitation, however, it is reasonable to infer that molecular mediators of primary resistance to trastuzumab-based therapy may also cause primary resistance to lapatinib. Among these, PI3K mutations seem to play a role in reducing the efficacy of both anti-HER2 drugs [19].

On the other hand, we observed that the duration of disease control with first line trastuzumab-based therapy correlated positively with both PFS and OS from the initiation of lapatinib and capecitabine. This suggests that, in tumors which initially respond to trastuzumab and then progress, resistance to trastuzumab is mediated by mechanisms that may be circumvented by EGFR/HER2 simultaneous tyrosine-kinase inhibition [20]. Unfortunately, the retrospective design of this study and the confounder related to the presence of chemotherapy, do not allow further speculation on the specific efficacy of lapatinib in patients who experience acquired resistance to trastuzumab after long-term exposure. This hypothesis, however, warrants clinical confirmation for its potential implications in the current positioning of treatment with lapatinib and capecitabine in the therapeutic sequence for patients with HER2-positive advanced breast cancer.

## Conclusion

In a real life setting, HER2 positive metastatic breast cancer patients treated with lapatinib and capecitabine after at least one regimen containing trastuzumab, achieved a median PFS of 7 months and a median OS of 26 months, which are consistent with the results of the prospective, randomized, phase 3 registration trial. Sensitivity to the first trastuzumab-based therapy both in term of tumor response and longer TTP were associated with longer PFS and OS with lapatinib and capecitabine, suggesting that lapatinib-based therapy may overcome acquired clinical resistance to trastuzumab in a proportion of patients.
